# Comprehensive bibliometric and visualized analysis of research on incisional hernia and component separation from 1996 to 2023

**DOI:** 10.1016/j.jpra.2025.04.012

**Published:** 2025-04-25

**Authors:** Mohamed Maatouk, Imen Maatouk, Ghassen Hamdi Kbir, Ons Hmandi, Mariem Nouira, Mohamed Ben Khalifa

**Affiliations:** a A21 Surgery Department, Charles Nicolle Hospital, Research laboratory LR12ES01, Faculty of medicine of Tunis, Tunis El Manar University, Tunis, Tunisia; b University of Monastir, Textile Engineering Laboratory (LGTex), Ksar-Hellal, Tunisia; c Department of Forensic Medicine, Charles Nicolle University Hospital, Tunis, Tunisia; d Faculty of Medicine, University of Tunis El Manar, Tunis, Tunisia; e Department of Medical Epidemiology, Charles Nicolle Hospital, Faculty of medicine of Tunis, Tunis El Manar University, Tunis, Tunisia; f General surgery Department Tahar sfar hospital Mahdia, Faculty of Medicine, University of Monastir Tunisia, Tunisia

**Keywords:** Bibliometric analysis, Incisional hernia -Component separation

## Abstract

**Introduction:**

In parallel with the increasing number of studies published on component separation technique (CST), there are no bibliometric studies on this subject. This study aimed to report previously published literature in CST to identify the research status.

**Methods:**

A search was undertaken for documents published between 1996 and 2023 from the PubMed, Web of Science, and Scopus databases, using the keywords “incisional hernia” and “component separation.”

**Results:**

A total of 469 publications was found from 121 different journals. The USA was in the leading position in several fields (productive authors, active institutions, and international cooperation).

CST was predominantly published in plastic surgery journals; however, after more specialized journals on topics such as *Hernia* became predominant, dominating the number of publications since 2013. “Transversus Abdominis Release” and “minimally invasive” have been trending keywords in recent years.

**Conclusion:**

Although CST research on incisional hernia repair has witnessed notable growth, publications in plastic surgery journals have declined. We encourage these journals to highlight the critical role of plastic surgery and the importance of collaboration between general and plastic surgeons worldwide, focusing on minimally invasive techniques to establish robust evidence for the standard repair of large abdominal wall defects.

## Introduction

Incisional hernia (IH) is a frequent complication following abdominal surgery, occurring in 3%-20% of cases after midline laparotomy.^1^ Achieving anatomical restoration of the linea alba, cannot be achieved in large abdominal wall defects, particularly in complex IH.^2^ Despite the availability of several reviews, there is no consensus regarding the most appropriate and effective technique for treating the defect. A review on the repair of complex IH found that the component separation technique (CST) with mesh repair may be advantageous and preferable in comparison to other surgical techniques.^3^ CST, introduced by Ramirez et al. in 1990, relies on enlarging the abdominal wall surface by separating and advancing the muscular layers to bridge defects up to 20 cm.^2^ Currently, CST is being increasingly applied and gaining popularity. In parallel with the increasing number of studies published on CST, there are no bibliometric studies on this condition. Thus, it would be beneficial to comprehend the existing scientific situation and direct future research to various fields of science.

In this regard, we conducted a bibliometric analysis combined with visualization tools utilizing the Scopus, Web of Science (WoS) Core Collection, and PubMed databases.

This study aimed to analyze previously published literature on CST to identify the research status and trends. This can help professionals to identify the key evidence and highlight knowledge gaps in the field of abdominal wall reconstruction, thus encouraging new research investigations to guide decision-making.

## Methods

### Source database and Search strategy

To cover as many target documents as possible, our analysis was conducted using the PubMed, WoS, and Scopus databases. The search keywords were defined by combining the terms “incisional hernia” and “component separation.” Table 1S shows the search queries used to export the papers. Databases were searched on March 27, 2024, which led to the elimination of articles published in 2024, because the year was incomplete. The relevant studies were published from January 1996 to December 2023. Documents such as guidelines, consensus, letters to the editor, scientific letters, and editorials were excluded. The inclusion of studies from 1996 onward was chosen because the CST, initially described by Ramirez et al. in 1990, began to gain widespread adoption in 1996.^4^^,^^5^

Two independent authors (MM and IM) separately reviewed the selected articles by keywords, titles, and abstracts to ensure their relevance. Any disagreements were discussed until consensus was achieved. Finally, the complete records were collected for bibliometric analysis.

### Data Collection

We performed manual screening to extract the following basic information from the selected studies: title, author names, publication’s year, country, institutional affiliations, number of citations, keywords, topic, and study types. The extracted data were saved in a .csv and .txt file and opened in Microsoft Excel 2019 (Microsoft Corporation, USA) for additional examination.

### Bibliometric and visualized analysis

In this bibliometric analysis, we used 2 distinct software: the Biblioshiny application of the bibliometrix, namely, the 4.3.2 package in R (Aria & Cuccurullo, 2017) for performance analysis and VOSviewer 1.6.20 (Leiden University, Leiden, The Netherlands) for network visualization.

The VOS viewer was used to analyze and visualize co-authorship networks created using authors, affiliations, journals, co-cited analysis of cited authors, and keyword occurrence.

Subsequently, a .txt format word list was created, with the data divided into 2 columns (labeled and replaced by), as advised by the VOS viewer user manual.

## Results

### Basic characteristics

**Figure 1:**
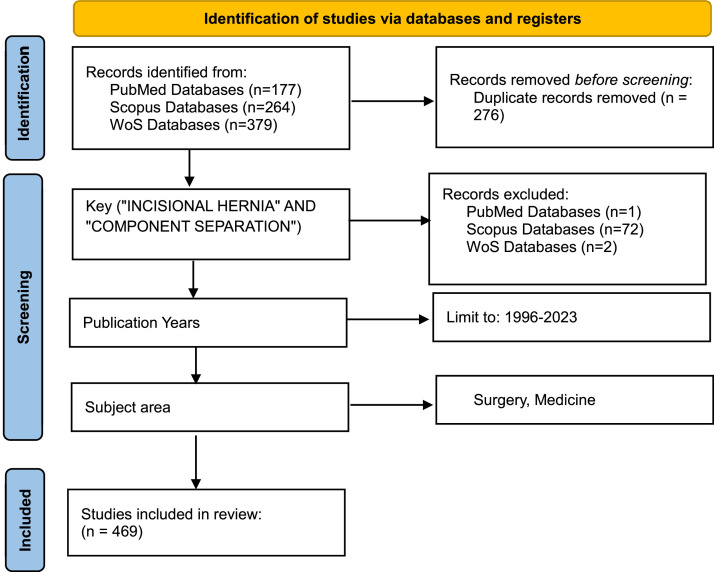
Prisma flow diagram.

As a result of the literature review, we identified 820 relevant studies (379 papers from the WoS database, 264 articles from the Scopus database, and 177 articles from the PubMed database). After removing duplicates and manual screening, 469 full-text studies from 121 different journals were selected. Figure 1 depicts the flowchart of selection.

The basic features of the included studies are presented in Table 1. The average annual rate of increase was 15.59%. A total of 1951 authors contributed to the field of CST in IH repair research. The overall number of citations was 5598 with an average citation rate of 11.94 per paper.

**Table 1:** Main data information.

Description	Results
**Main information about data**	
Timespan	1996 to 2023
(28 years)	
Sources (journals, books, etc)	121
Documents	469
Annual growth rate (%)	15.59
Document average age (years)	6.65
Average citations per doc	11.94
References	3973
**Document contents**	
Keywords plus (ID)	1348
Author's keywords (DE)	1466
**Authors**	
Authors	1951
Authors of single-authored docs	13
**Authors collaboration**	
Single-authored docs	17
Co-authors per doc	5.66
International co-authorships (%)	7676

### Distribution of articles by years of publication

**Figure 2:**
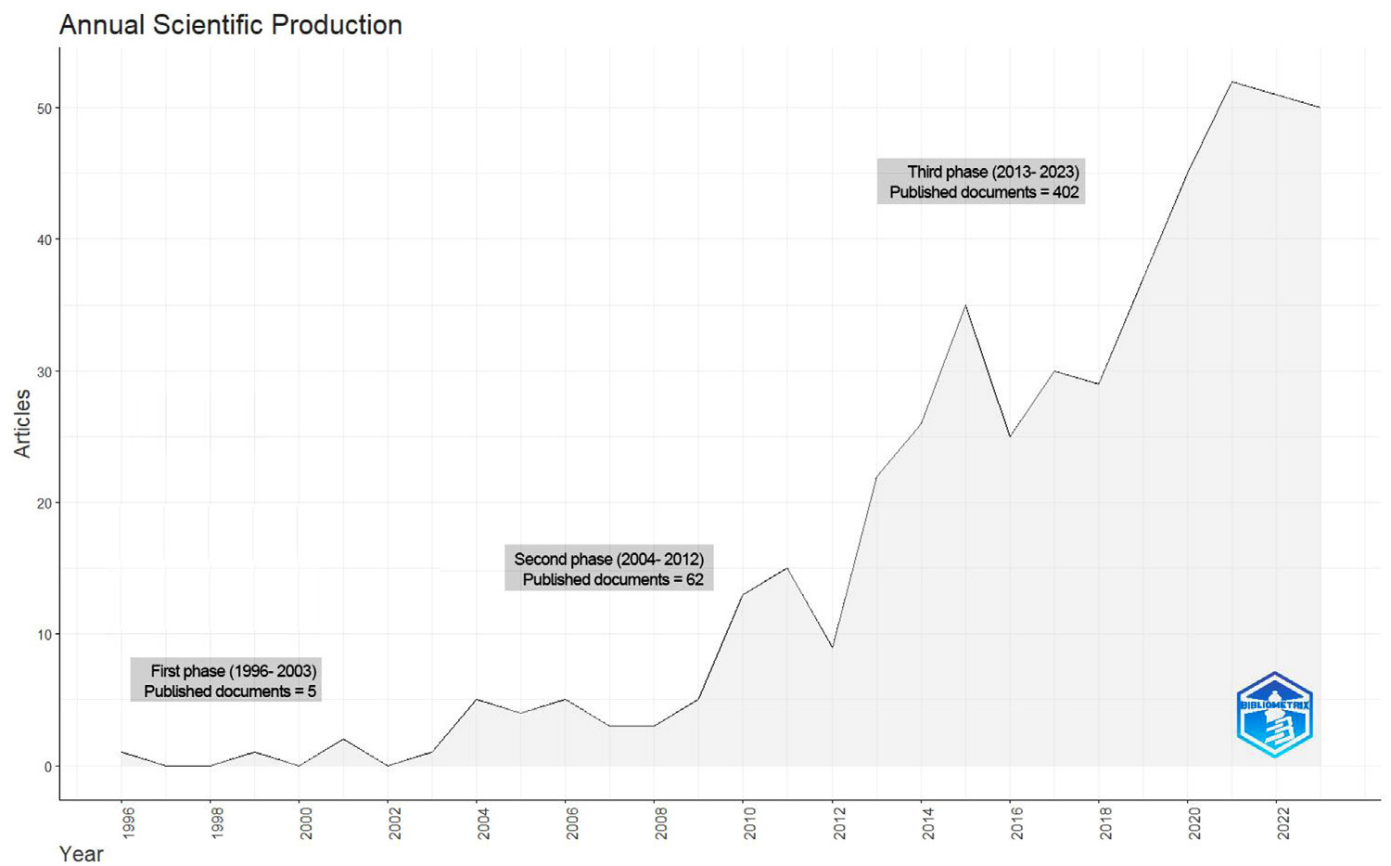
Annual scientific production on incisional hernia and component separation (1996-2023).

Figure 2 shows the progression of relevant published articles between 1996 and 2023. The number of published papers has steadily increased, from 1 in 1996 to 50 by 2023. The research period can be divided into 3 phases: Phase one (1996-2003) with low production, phase 2 (2004-2012) with moderate production, and phase 3 (2013-2023) with high production. Approximately 85% of the papers have been published since 2013 with 2021 being the most productive year with 52 papers, followed by 2022 and 2023 with 51 and 50 articles, respectively.

### Distribution of articles by country and institution

**Fig 3:**
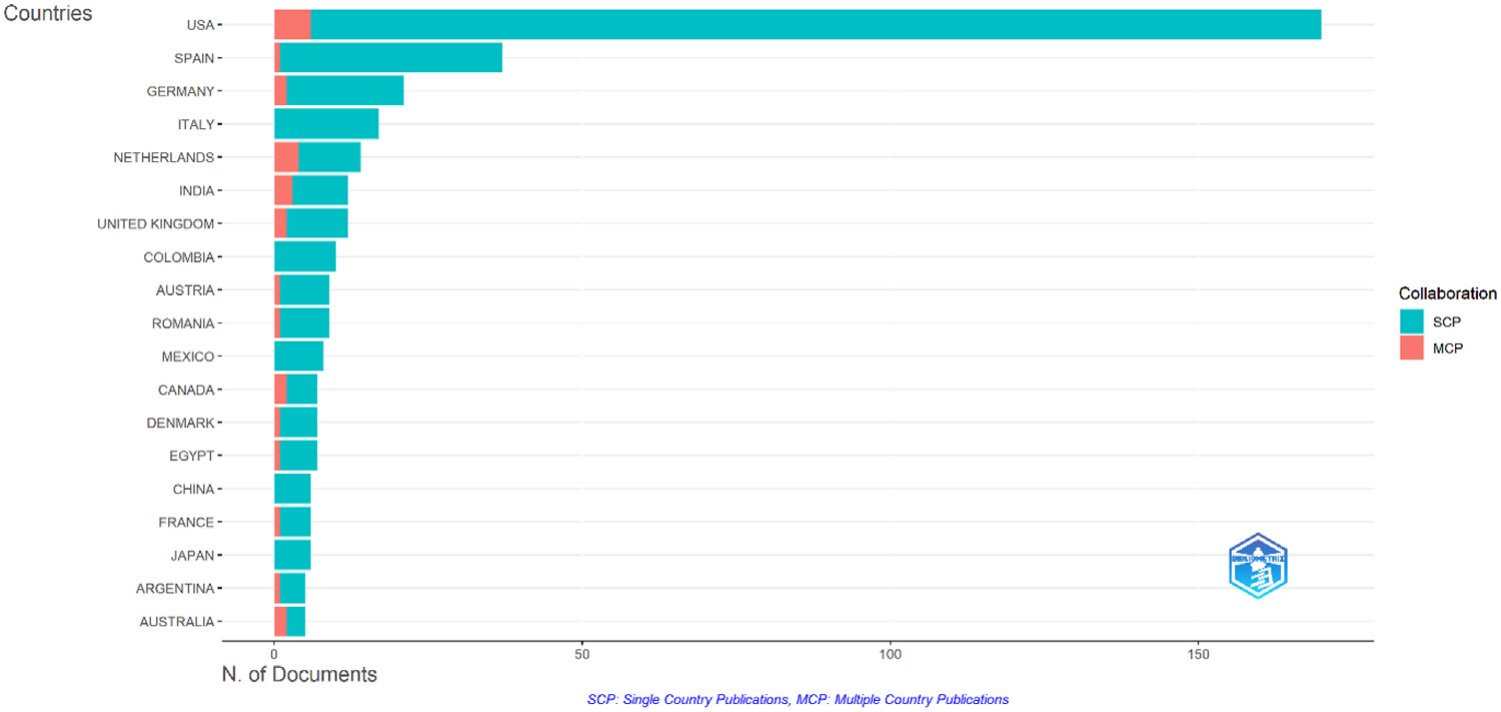
Distribution of the number of articles by country.

The distribution of the number of articles by country is illustrated in Figure 3. The USA contributed the greatest number of publications with 235 articles (50.1%), followed by Spain and Germany with 37 (7.8%) and 21 (4.4%) articles. The top 3 institutions with the most productive articles were Carolinas Medical Center with 31 articles (6.6%), University of Kentucky with 22 articles (4.7%), and Washington University with 20 articles (4.7%).

**Fig 4:**
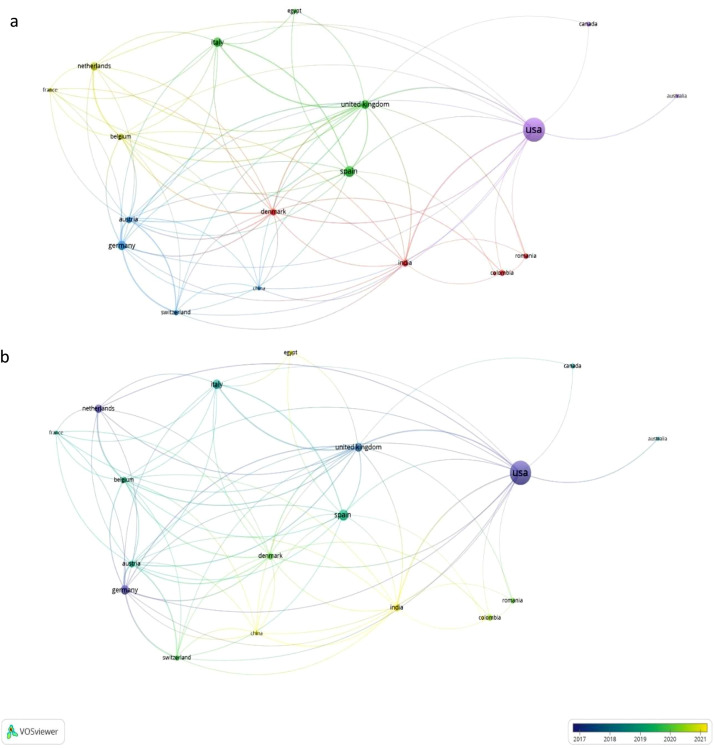
Country analysis. a- Network visualization map of co-authorship (International collaboration). The size of the circle represents the scope of international collaboration. b- Overlay visualization map showing the trends of country frequency over time.

According to the results of the cluster analysis (Figure 4a), 5 different clusters related to international cooperation were formed. The main cluster was prominently constituted by USA, Canada, and Australia. The second largest cluster was formed by Spain, the United Kingdom, Italy, and Egypt.

Figure 4b illustrates the overlay of the trends in the frequency of country relationships over time, highlighting countries such as India, China, and Colombia, which have shown a gradual increase in influence since 2021.

### Analysis of journals

The 10 journals with the most publications are listed in Table 2. *Hernia* had the most articles published on CST, totaling 67 articles. This journal produced 14.2% of all publications during the study period and 12.6% of the total citations (710 out of 5598). *Revista Hispanoamericana de Hernia* ranked second with 27 articles, followed by *Surgical Endoscopy and Other Interventional Techniques*, which published 26 papers. *Plastic and Reconstructive Surgery* and *Annals of Plastic Surgery* were the oldest journals publishing papers on this topic. Figure 1S illustrates the production evolution of the 5 most productive sources involving the theme of IH and component separation. *The American Journal of Surgery* ranked first in terms of publications from 2007 to 2013. After 2013, journals focusing on the specific research field of abdominal wall reconstruction, such as *Hernia* and *Revista Hispanoamericana de Hernia*, took first place in the number of publications.

**Table 2:** Top 10 journals with the most documents.

Element	h_index	NP	TC	PY_start
*Hernia*	16	67	710	2004
*Revista Hispanoamericana De Hernia*	3	27	26	2013
*Surgical Endoscopy And Other Interventional Techniques*	13	26	836	2011
*American Journal Of Surgery*	11	20	781	2006
*Plastic And Reconstructive Surgery*	12	14	433	1996
*Annals Of Plastic Surgery*	9	17	367	1999
*Surgery*	9	15	234	2014
*American Surgeon*	7	12	164	2011
*Annals Of Surgery*	6	6	456	2015
*Journal Of The American College Of Surgeons*	5	7	284	2003

NP: number of publications, TC: total citations, PY: publication year

### Analysis of authors and citations

B Todd Heniford at the Carolinas Medical Center was the author with the highest number of publications, having published 15 articles (3.19%), followed by Rosen M., Fischer J., and Kercher K. with 13, 12, and 12 articles, respectively. In Table 3, we listed the top 10 authors with the most documents. Nine of the 10 authors with the most publications were from the United States. The authors with the highest number of citations were Novitsky et al. (875, 15.6%), followed by Roth J (514, 9.1%). Figure 2S elaborates on the top 10 authors’ productivity over time. Notably, 2021 saw a substantial increase in contributions. Novitsky et al., one of the most influential scholars, began publishing in 2011 and continued until 2018, producing highly cited articles (darker node). Moreover, most of the top 10 authors have continued to publish through 2023, indicating that this topic remains a current trend. Table 4 lists the top 10 most‑cited articles. The top-cited article was published by Novitsky et al. in the *American Journal of Surgery* and generated a total of 383 citations. Articles from USA accounted for all of the 10 most cited papers.

**Table 3:** Ten authors with the highest production of articles.

Author	Affiliation	Number of publications	Number of citations	PY start
Heniford B	Carolinas Medical Center	15	262	2014
Rosen M	Minimally invasive Surgery Center, Cleveland	13	367	2007
Fischer J	University of Pennsylvania Health System, Philadelphia,	12	142	2014
Kercher K	Carolinas Medical Center	12	229	2014
Augenstein V	Carolinas Medical Center	11	207	2015
Roth J	East Carolina University	10	514	2007
Novitsky Y	University of Connecticut Health Center, Farmington	9	875	2011
Colavita P	Carolinas Medical Center	9	169	2019
Berrevoet F	Ghent University Hospital, Belgium	8	40	2010
Petro C	University Hospitals Case Medical Center, Cleveland	8	91	2014

PY: publication year

**Table 4:** Top 10 most‑cited papers in the field of component separation for incisional hernia.

Rank	Title	Author	Journal	Year	NC	NC per year
1	Transversus abdominis muscle release: a novel approach to posterior component separation during complex abdominal wall reconstruction	Yuri W Novitsky et al.	*The American Journal of Surgery*	2012	383	29.4
2	Ventral Hernia Management: Expert Consensus Guided by Systematic Review	Mike K Liang et al.	*Annals of Surgery*	2017	218	27.2
3	A novel approach using the enhanced-view totally extraperitoneal (eTEP) technique for laparoscopic retromuscular hernia repair	Igor Belyansky et al.	*Surgical Endoscopy*	2018	187	26.7
4	Outcomes of Posterior Component Separation With Transversus Abdominis Muscle Release and Synthetic Mesh Sublay Reinforcement	Yuri W Novitsky et al.	*Annals of Surgery*	2016	136	15.1
5	Open ventral hernia repair with component separation	Eric M. Pauli MD et al.	*Surgical Clinics of North America*	2013	127	10.5
6	Primary fascial closure with mesh reinforcement is superior to bridged mesh repair for abdominal wall reconstruction	Justin H Booth et al.	*Journal of the American College of Surgeons*	2013	125	10.4
7	Multilayer reconstruction of abdominal wall defects with acellular dermal allograft (AlloDerm) and component separation	Adam R Kolker et al.	*Annals of Plastic Surgery*	2005	114	5.7
8	Does Mesh Location Matter in Abdominal Wall Reconstruction? A Systematic Review of the Literature and a Summary of Recommendations	Frank P Albino et al.	*Plastic and Reconstructive Surgery*	2013	104	8.6
9	SAGES guidelines for laparoscopic ventral hernia repair	Earle D et al.	*Surgical endoscopy*	2017	93	10.3
10	Comparative analysis of open and robotic transversus abdominis release for ventral hernia repair	Bittner JG et al.	*Surgical endoscopy*	2018	91	13

NC: number of citations

### Distribution of keywords

**Fig 5:**
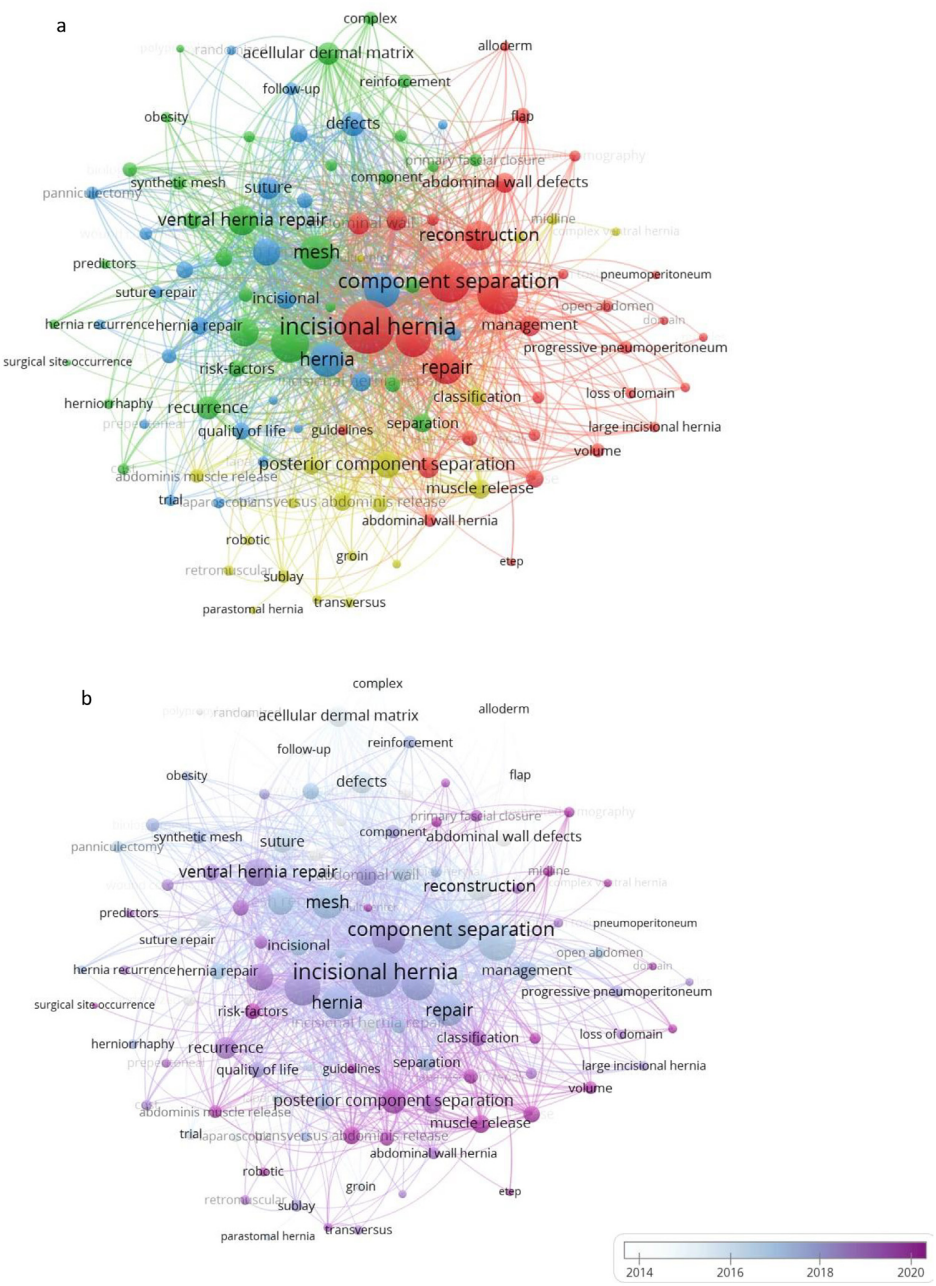
a- Network visualization map of keywords. b- Overlay visualization map shows the trend of high-frequency keywords over time.

Among the 1337 terms/keywords retrieved from the 496 publications, 111 words were represented more than 10 times. The most frequent keyword was “incisional hernia” with 254 occurrences, followed by “hernia” (118), “closure” (100), and “surgical mesh” (94). In the cluster analysis map (Figure 5a), keywords are classified into 4 clusters. Each cluster represents keywords commonly used together. Keywords in red pertain to specific technical artifices used in the treatment of ventral hernias with loss of domain (pneumoperitoneum, botulinum toxin, alloderm, and flap). The second and third cluster in blue and green indicates the outcomes of CST (recurrence, risk factors, quality of life, infection, and wound complications). Finally, the yellow cluster addresses the techniques of posterior component separation and surgical approach (transversus abdominis muscle release, laparoscopic, and robotic). Through the time span of the keyword relationships (Figure 5b), more attention has been paid in recent years to minimally invasive posterior component separation. Figure 3S clearly demonstrates that the topics “Transversus Abdominis Release,” “TAR,” and “anterior component separation” have gained popularity.

## Discussion

Despite innovations in surgical techniques, the incidence and costs of IH repair have increased. Each IH repair cost €4153 in hospital care in France. The annual cost increase in the USA is estimated to be 74.6%.^6^^,^^7^ Therefore, IH represents a major healthcare burden. In a bibliometric study conducted on IHs published from 2003 to 2023, Xv et al.^1^ reported 10,504 articles. The top 100 most cited articles were analyzed and CST was not identified as one of the most highly cited keywords. However, CST has been applied increasingly in the treatment of IH, particularly for complex and large abdominal wall defects. Currently, the separation technique is the procedure of choice for large IH repair because of its potential advantages in reducing the incidence of surgical site occurrences. Therefore, there has been an increasing interest in performing CST.^8^ To the best of our knowledge, this is the first bibliometric and visualized study to evaluate the characteristics of the scientific production of articles on CST in IH repair. The analysis revealed an ongoing increase in the scientific production on this topic, with the USA ranking first globally with 235 publications, representing 50.1% of all included articles.

Given the considerable financial resources allocated for research, large number of research centers, and increasing adoption of CST in the USA, this finding is unsurprising. In his study on the trends in surgical techniques for IH repair in the USA, Howard et al. stated that the use of the minimally invasive CST increased from 1.8% in 2007 to 16.3% in 2018.^9^ Similarly, bibliometric studies on inguinal and IH repair indicated that the USA was a pioneering country in terms of research productivity.^10^ Moreover, investigators and institutions in the USA have dominated this research topic. Consequently, USA plays a significant role in the field of abdominal wall reconstruction. Surgeons worldwide have frequently adopted recommendations and guidelines established by the American societies. For instance, the Society of American Gastrointestinal Endoscopic Surgeons established scientifically supported guidelines for managing laparoscopic ventral hernia repair. These recommendations rank among the most frequently cited publications on CST in IH repair and have received a total of 185 citations (Table 2).

Moreover, enhancing international collaborations could support a more globally diverse research environment; initiating collaborative projects, project applications, and academic exchanges; and offering information that can be used to improve patient outcomes in different healthcare systems.

Furthermore, universities and research institutions in the USA should be encouraged to provide funding and resources to support research programs in lower-income countries. Therefore, we could help introduce CST techniques to reduce global health disparities in IH management.

In the correlation analysis, it was found that since 2021, increasing cooperation between India, China, and Colombia has led to new strategic alliances impacting global cooperation and competition. Given their dominant position in CST publications, it is crucial for these countries to collaborate with the USA to leverage their expertise and leadership in advancing research and techniques. This collaboration can significantly advance scientific research and drive the economies of these developing countries, particularly in addressing the high population densities of India and China.

The present study highlights the superiority of journals that focus on specialized surgical topics in the field of abdominal wall reconstruction. *Hernia* and *Revista Hispanoamericana de Hernia* are the most productive journals. However, none of the top 10 articles were published in these journals. Most journals publishing top-cited articles, such as *Surgical Endoscopy* and *Annals of Surgery*, are widely recognized and influential. This can be explained by the fact that these specialized journals began publishing more frequently after 2015, resulting in fewer citations. Another finding of the study is that before 2012, CST was predominantly published in plastic surgery journals, whereas after 2012, more specialized journals on hernia topics became predominant. This is understandable when considering that Mr. Ramirez, the first person to define the surgical technique of component separation, was a plastic surgeon. His study published in *Plastic and Reconstructive Surgery* in 1990 was the landmark of component separation.

Over time, the treatment of ventral hernias using CST has increasingly been performed by general surgeons rather than plastic surgeons. This may be explained by the increasing popularity of laparoscopy and robotics in wall abdominal reconstruction. To date, few studies have evaluated the outcomes of IH with CST by surgical specialty. Using a large-scale national database, Reid et al.^11^ demonstrated that open ventral hernia repair is most commonly completed by a general surgeon (53,282 [99.1%]) than a plastic surgeon (464 [0.9%]). However, in the case of severe or complex IH, plastic surgeons are involved in performing adjunctive procedures such as CST (24.8% vs. 5.3%, P < 0.001) or acellular dermal matrix. In addition, complication rates were found to be lower in patients whose repairs involved plastic surgeons. Similar published reports^12^^,^^13^ have supported these results and indicated that patients operated on by plastic surgeons had more complex defects and higher abdominal pressure. Moreover, Chang et al.^14^ found that a concomitant panniculectomy could be performed safely by plastic surgeon in conjunction with the ventral hernia repair with CST in patients with class III obesity. In fact, plastic surgeons are more experienced in handling complex abdominal wall repairs due to their specialized training, which focuses on adequate tissue handling, containing the design and transfer of flaps, and a detailed understanding of the anatomy and vascularization of the anterior abdominal wall.

Considering these reasons, we recommend an interdisciplinary collaboration between general and plastic surgeons. Furthermore, concomitant techniques such as perforator-sparing flaps, acellular dermal matrix, and panniculectomy highlight the critical role of plastic surgery in complex IH treatment.

Therefore, we encourage plastic surgery journals to continue publishing on this subject, as plastic surgeons will remain key contributors to the evolution of CST, and their collaboration will be essential in advancing the technique and outcomes in abdominal wall reconstruction.

Ramirez et al. described the classic anterior CST in which the external oblique was vertically incised and separated from the internal oblique.^4^ Later, in 2012 Novitsky et al.^15^ described a novel technique of posterior CST, the transversus abdominis release. This article received the highest number of citations in our bibliometric analysis .Rapidly, this technique is emerging as the preferred method for myofascial advancement in complex ventral hernia repair. Hence, the increase in the popularity of CST for IH repair is reported in the study. These 2 publications (Ramirez et al. 1990 and Novitsky et al. 2012) were the top 2 cited authors in reference as shown in the network visualization of co-citation analysis of authors (Figure 4S), and could be considered as the summary of component separation.

Regarding the co-occurrence network of terms, “minimally invasive,” “posterior component separation,” and “TAR” have become more frequent, demonstrating its current status as the preferred approach for complex IH treatment worldwide. Our findings are supported by those of previous studies that have well documented the benefits of the minimally invasive approach compared to the open approach.^16^^,^^17^ In a recent meta-analysis, Tryliskyy et al. reported lower rates of wound-related risk and overall complications.^8^ In 2017, the guidelines recommended giving preference to minimally invasive CST over open CST.^18^

We acknowledge some potential limitations in our study. First, only studies published in English were analyzed. Although English is the most frequently used language for publication, some publications in other languages may have been missed.

This language restriction could have caused an under representation of research conducted in non-English-speaking countries, potentially biasing the geographical distribution of the contributions. Future studies could incorporate multilingual databases, including non-English publications, particularly those in Chinese, to provide a more comprehensive overview, especially considering China's recent growing interest in CST.

Second, the keywords that we used in our search “incisional hernia” and “component separation” appeared to be restrictive. We included these specific terms to ensure that our search was highly targeted.

Third, data were only retrieved from the PubMed, WoS, and Scopus databases and did not include other medical databases such as Google Scholar.

Fourth, our study focused primarily on the volume of publication and citation analysis, which may not fully reflect the clinical impact or quality of the research. Future studies could incorporate qualitative assessments, to provide a more comprehensive understanding of the clinical significance and practical implications of CST advancements.

Despite these facts, this is the first bibliometric analysis of articles in the field of CST and clearly highlight the contributors and research interests. Our study will potentially indicate possible directions for scientists to develop guidelines and best practices for the management of complex IH by revealing current trends and new approaches. Clinical guidelines could incorporate the results of our analysis to support the development and use of the posterior CST, the benefits of new minimally invasive approaches, and provide valuable recommendation for their application.

Further research is needed in this field to enhance the evidence base for the minimally invasive CST techniques, by comparing minimally invasive and open approaches and prioritizing long-term outcomes and quality of life. As mentioned earlier, these studies should be conducted through international collaboration to stimulate the development of innovative techniques and reduce disparities between counties.

## Conclusion

Since its first description, there is an expanding body of literature describing the CST in IH repair. This analysis showed that the papers were predominantly authored by researchers from the USA. However, with the recent interest from China and India, countries that are experiencing significant population growth and economic development, it is crucial to enhance support for studies in this field, particularly within these 2 countries. Additionally, fostering collaboration between institutions in the USA and other developing countries is essential to advance research and innovation in this area. Furthermore, there is a need to encourage journals specializing in abdominal wall reconstruction research to enhance their impact factor, thereby amplifying the influence and reach of this trend.

Notably, there has been a decline in publications on this subject in plastic surgery journals, and we encourage these journals to continue highlighting the importance of collaboration between general and plastic surgery, as well as the key role of plastic surgeons, whose expertise in tissue handling, flap design, and soft tissue repair is essential for the success of complex abdominal wall reconstructions.

This study could be used to guide future research, offering valuable insights for clinicians, surgical assistants, and health policymakers, as it emphasizes the growing interest and emerging prominence of minimally invasive techniques in CST. Additional high-quality studies could be designed to assess the safety and advantages of minimally invasive CST, providing robust evidence to recommend as the reference standard for the repair of large abdominal wall defects.

## Conflict of Interest

The authors declare that they have no conflict of interest.

## Appendix Supplementary materials

Image, application 1
