# Supplementary material for: Comprehensive bibliometric and visualized analysis of research on incisional hernia and component separation from 1996 to 2023
Source: JPRAS Open. 2025 Apr 25;45:76–88. doi: 10.1016/j.jpra.2025.04.012 (PMC12166838; doi:10.1016/j.jpra.2025.04.012)
Supplement: Supplementary file 1 [file mmc1.docx]

| Database | Query |
| --- | --- |
| PubMed | "Incisional hernia"[All Fields] AND "component separation"[All Fields] AND "english"[Language] AND "journal article"[Publication Type] AND 1990/01/01:2023/12/31[Date - Publication] |
| Web of science | "INCISIONAL HERNIA" AND "COMPONENT SEPARATION" (Topic) and 2024 (Exclude – Publication Years) and Article (Document Types) and Surgery (Research Areas) |
| Scopus | TITLE-ABS-KEY ( "INCISIONAL HERNIA" AND "COMPONENT SEPARATION" ) AND PUBYEAR > 1995 AND PUBYEAR < 2024 AND ( LIMIT-TO ( SUBJAREA , "MEDI" ) ) AND ( LIMIT-TO ( DOCTYPE , "ar" ) ) |

**Table 1S** :Document search query


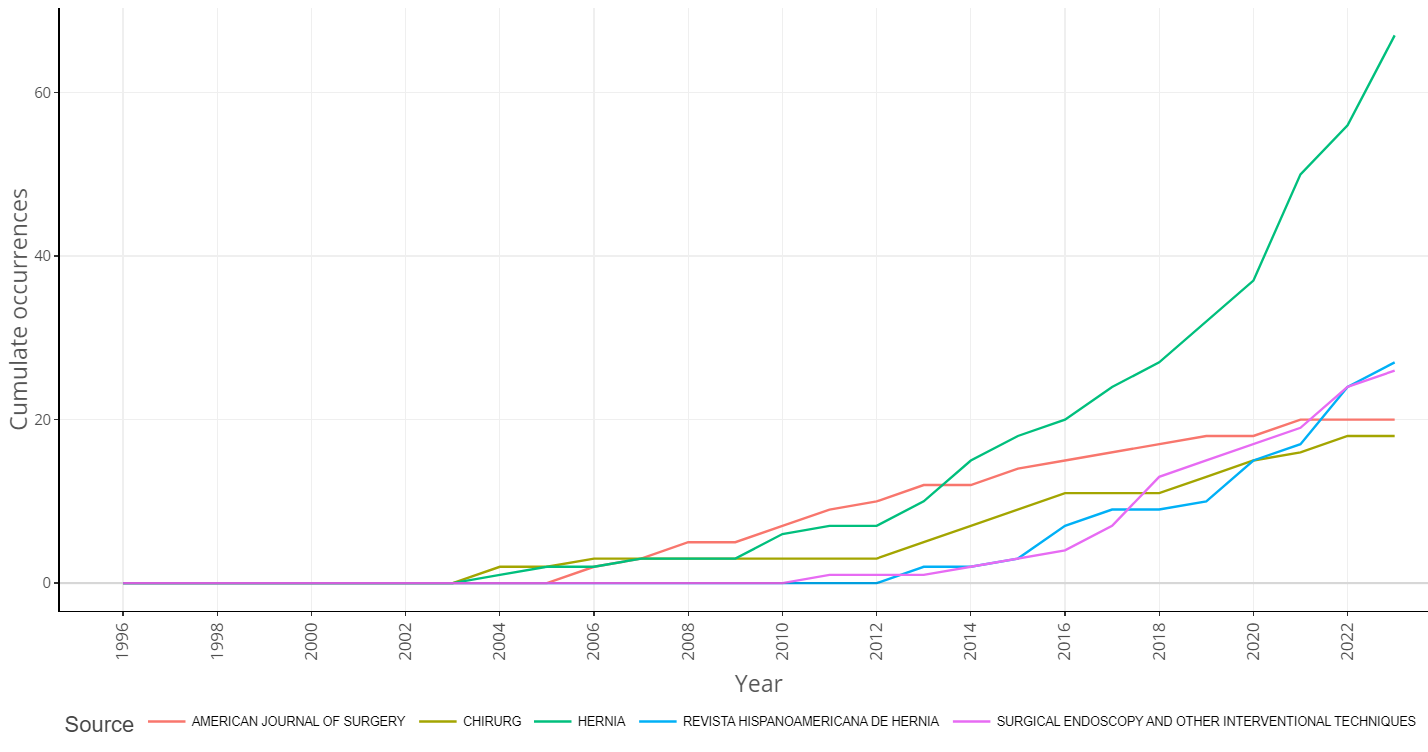


**Figure 1S:** Production evolution of the five most productive sources


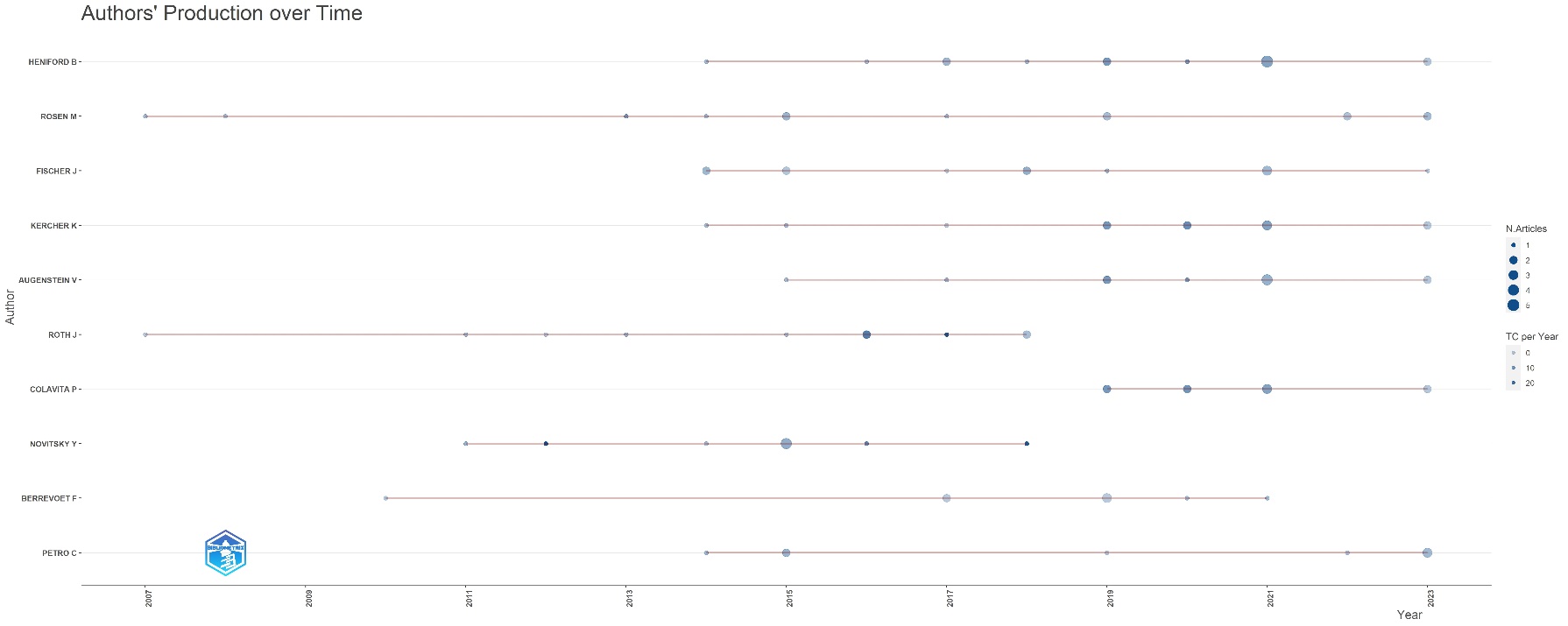


**Fig 2S:** The top 10 author’s production over time


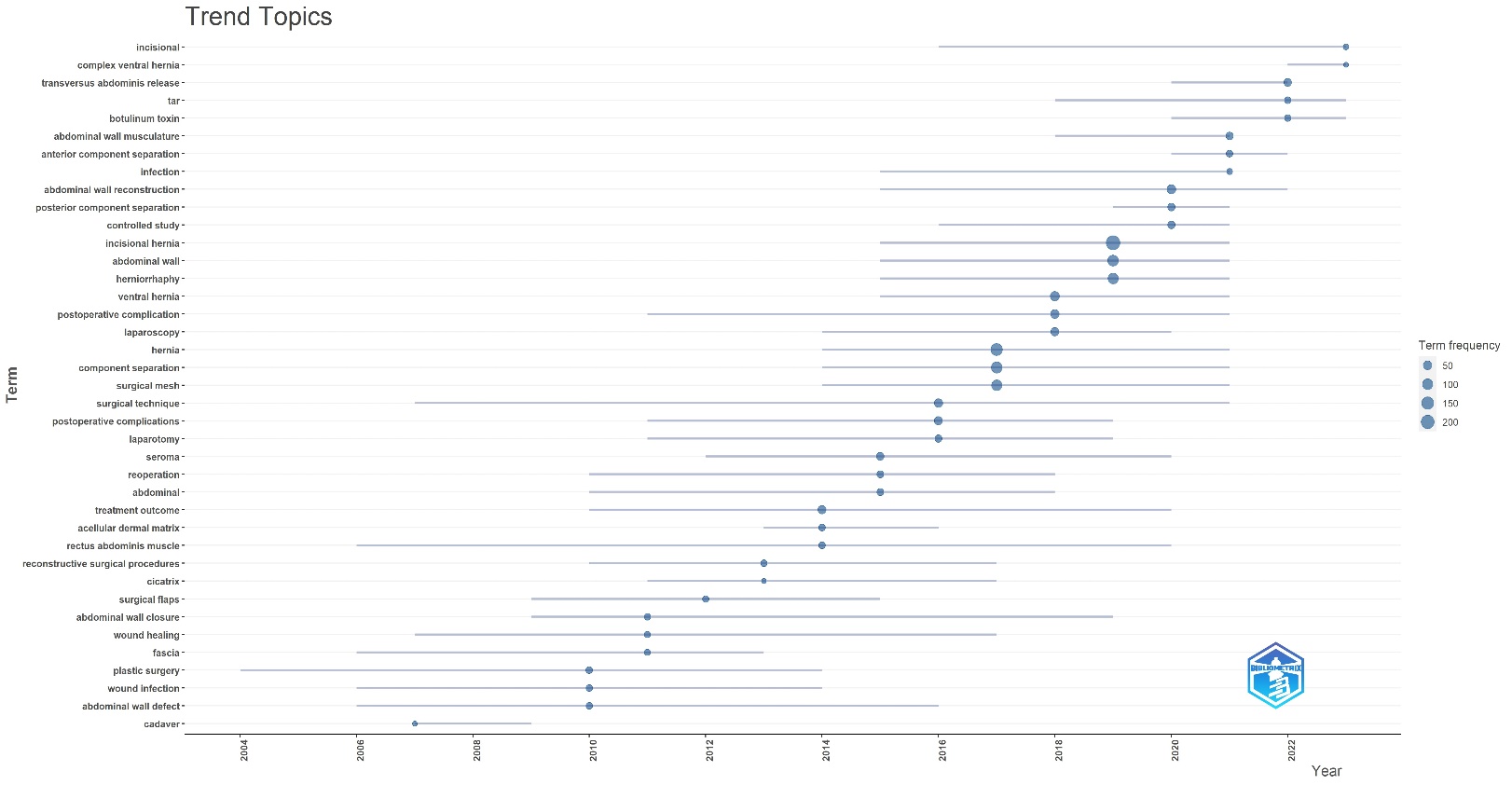


**Fig 3S:** Trend topics


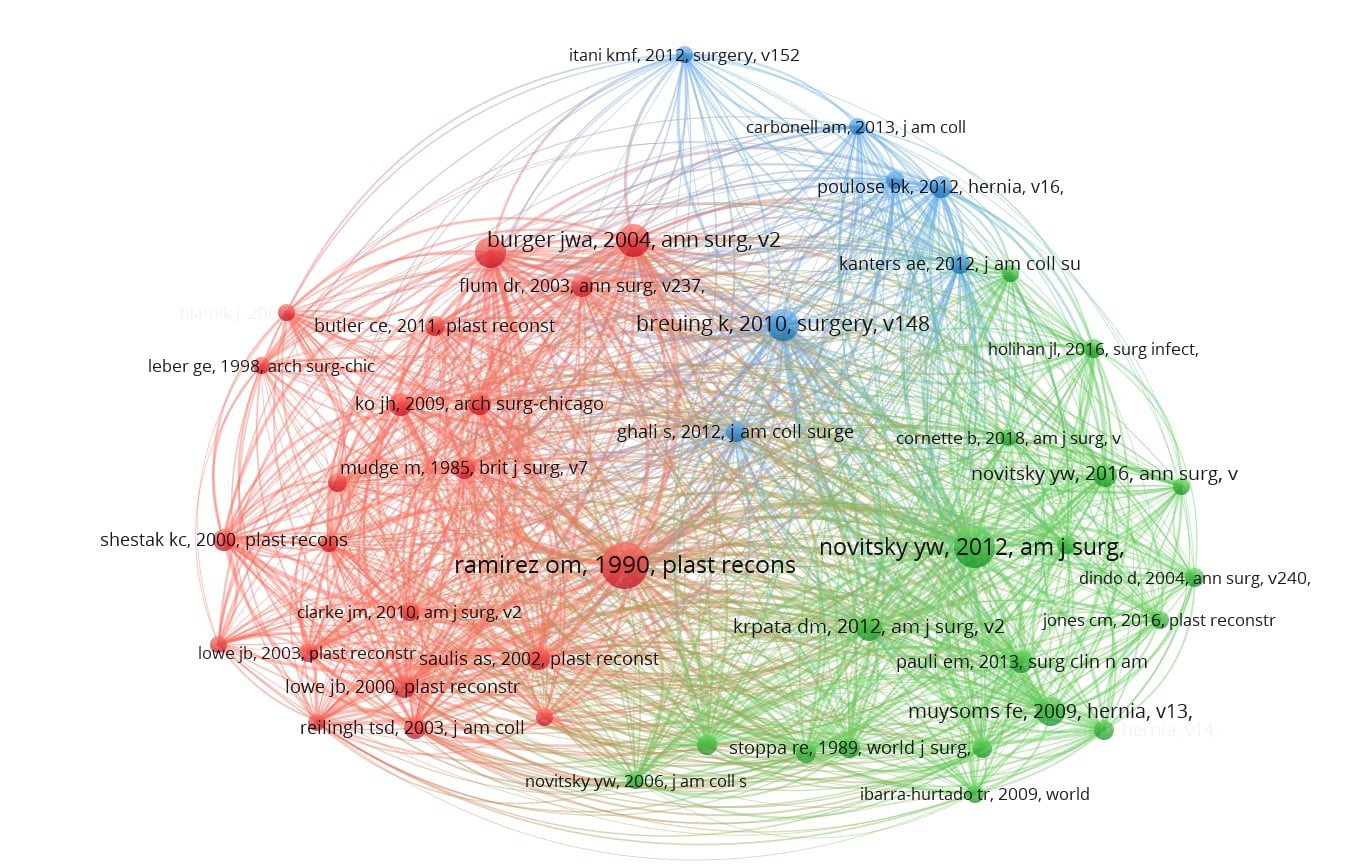


**Fig 4S**: the network visualization of co-citation analysis of authors
